# The impact of the inpatient practice of continuous deep sedation until death on healthcare professionals’ emotional well-being: a systematic review

**DOI:** 10.1186/s12904-017-0205-0

**Published:** 2017-05-08

**Authors:** Sarah Ziegler, Hannes Merker, Margareta Schmid, Milo A. Puhan

**Affiliations:** Epidemiology, Biostatistics and Prevention Institute, University of Zurich, Hirschengraben 84, CH-8001 Zurich, Switzerland

**Keywords:** End-of-life care, Terminal care, Sedation, Well-being, Stress, Nurse, Physician

## Abstract

**Background:**

The practice of continuous deep sedation is a challenging clinical intervention with demanding clinical and ethical decision-making. Though current research indicates that healthcare professionals’ involvement in such decisions is associated with emotional stress, little is known about sedation-related emotional burden. This study aims to systematically review the evidence on the impact of the inpatient practice of continuous deep sedation until death on healthcare professionals’ emotional well-being.

**Methods:**

A systematic review of literature published between January 1990 and October 2016 was performed following a predefined protocol. MEDLINE, EMBASE, PubMed, Cochrane Library, CINAHL, Scopus, and PsycINFO were searched using search terms within “end-of-life care”, “sedation”, and “emotional well-being”. Dissertations and reference lists were screened by hand. Two independent reviewers conducted study selection, data extraction and quality assessment. We abstracted measures of psychological outcomes, which were related to the practice of continuous deep sedation until death, including emotional well-being, stress and exhaustion. We used the GRADE approach to rate the quality of evidence.

**Results:**

Three studies remained out of 528 publications identified. A total of 3′900 healthcare professionals (82% nurses, 18% physicians) from Japan (*n* = 3384) and the Netherlands (*n* = 16) were included. The prevalence of sedation-related burden in nurses varied from 11 to 26%, depending on outcome measure. Physicians showed medium levels of emotional exhaustion and low levels of depersonalization. Common clinical concerns contributing to professionals’ burden were diagnosing refractory symptoms and sedation in the context of possibly life-shortening decisions. Non-clinical challenges included conflicting wishes between patients and families, disagreements within the care team, and insufficient professionals’ skills and coping. Due to the limited results and heterogeneity in outcome measure, the GRADE ratings for the quality of evidence were low.

**Conclusions:**

Current evidence does not suggest that practicing continuous deep sedation is generally associated with lower emotional well-being of healthcare professionals. Higher emotional burden seems more likely when professionals struggled with clinical and ethical justifications for continuous deep sedation. This appeared to be in part a function of clinical experience. Further research is needed to strengthen this evidence, as it is likely that additional studies will change the current evidence base.

## Background

Despite the substantial progress in medical care, patients can still experience unbearable suffering. When all standard therapies have failed and no alternative for palliation is available, palliative sedation can be used to treat refractory symptoms including pain, delirium, and dyspnoea [1]. The level of sedation varies in duration and depth from intermittently to continuously and mild to deep [2]. Continuous deep sedation until death (CDS) is only indicated for terminally ill patients with a life expectancy no longer than days or hours [3]. According to current guidelines, benzodiazepines are medications of first choice [4]. The use of opioids for sedation is contraindicated and therefore considered inappropriate [1, 5]. Abuse of palliative sedation occurs when death hastening is intended [1, 6].

The prevalence of CDS varies considerably between countries, healthcare settings, and patient populations. European nationwide studies have estimated the prevalence to be between 2.5 and 17.5% with the trend increasing over time [7–11]. Recent findings from Switzerland have pointed to a threefold increase from 4.7% in 2001 to 17.5% in 2013 of all deaths [11]. In contrast decreasing trends have been shown in Belgium from 14.5% in 2007 to 12.0% in 2013 [9]. These variability in estimates are partly due to lack of common CDS definition stemming from different values and concerns that are a function of different cultural backgrounds [12, 13].

There is evidence that patient’s suffering of unmanageable symptoms is a stressor for professionals’ emotional exhaustion [14]. Caring for a dying patient is potentially burdensome and leads to emotional exhaustion in every third US physician [15]. Nationwide Japanese data reveal that 15% of palliative care physicians are emotionally exhausted [16]. One of the highest prevalence of emotional exhaustion in palliative care clinicians was found in US hospice and palliative care with 59.5% reporting high levels of emotional exhaustion [17].

The case of CDS clinical intervention is particularly demanding, as decision-making is based on both clinical indications and complex non-clinical factors [18, 19]. Healthcare professionals are challenged with the assessment of unbearable suffering and refractoriness and furthermore with predicting life expectancy [20]. This is even more difficult when patients are no longer able to communicate as symptom intolerability is largely determined by patients [21]. Sixty tree percent of Norwegian nurses have reported ethical problems with CDS when patients were not able to express their wishes and suffering [22].

Evaluating clinical justification for CDS is especially delicate for patients with a life expectancy longer than a few days [23]. The timing of sedation administration and a lack of a common ethical framework can blur the line between end-of-life comfort and life-shortening decisions [24]. By definition CDS cannot be used with the intention of hastening death. Lack of a clear distinction seems rather to be due to imprecise understanding of the purpose of CDS and inadequate training in palliative care. To date there is no empirical evidence for shortened survival times among CDS patients [25, 26].

Besides clinical indications, the decision to start CDS is guided by personal wishes of individual patients and families as well as physicians’ experience and values [27–29]. However, engaging the family involves managing the emotional context of families and patients coping with suffering [28]. Conflicting wishes between patients and families and lack of consensus between healthcare professionals are potentially burdensome [20]. Longitudinal data have shown that dissatisfaction with teamwork can lead to professionals’ emotional exhaustion. Vice versa emotionally exhausted clinicians are less able to engage in multidisciplinary teamwork what further predicts clinician-rated patient safety [30].

To date, no systematic review focusing on the association between practicing CDS and healthcare professionals’ emotional well-being exists. Therefore, we aimed to systematically review evidence on the effect of the inpatient practice of CDS on the emotional well-being of healthcare professionals closest to CDS decisions and administration. We also investigated whether certain clinical and non-clinical factors contribute to CDS-related burden.

## Methods

The methods we used for this systematic review were based on the Centre for Reviews and Dissemination’s guidance for undertaking reviews [31]. The reporting followed the Preferred Reporting Items for Systematic Reviews and Meta-Analysis (PRISMA) [32].

### Data sources and search strategy

Per study protocol we performed a literature search in seven databases for studies published between January 1990 and October 2016. We conducted the initial search in MEDLINE and EMBASE and adapted the search strategy to PubMed - searching for literature in process - Cochrane Library, CINAHL, Scopus and PsycINFO. We hand-searched citations of relevant dissertations and reference lists of included studies.

The search strategy included controlled vocabulary terms and relevant free text words within the topics of “end-of-life care”, “sedation” and “emotional well-being”. There was no language restriction. For complete search strategy see Appendix 1.

### Study selection

#### We used the following inclusion criteria:

(i) Randomized controlled trials, comparative studies, cross-sectional and longitudinal quantitative studies, qualitative studies, cross-sectional and longitudinal mixed-methods studies; (ii) including a definition of CDS regardless of terminology but containing deep and continuous administration of sedatives until death for patients suffering from refractory symptoms for whom death is anticipated in the near future without intention to hasten death; (iii) patients population of terminally ill adults (≥18 years age); (iv) sedation in hospital, hospice, palliative care unit, or cancer center; (v) studies analysing healthcare professionals’ emotional well-being as primary or secondary endpoint including any psychological health related outcome assessed through medical records, fully or semi-structured questionnaires or interviews, or focus groups; (vi) healthcare professionals practicing CDS limited to general practitioners, physician assistants, physician associates, nurses, nurse practitioners, palliative care specialists, oncologists, or specialized palliative care nurses.

#### Studies were excluded if:

(i) the article was described as a case control study, review article, editorial, comment, letter or newspaper article; (ii) the study addressed sedation in context other than palliation, sedation not at the end-of-life and not to unconsciousness; (iii) the study addressed sedation within home care and nursing home; (iv) the study used surrogate outcomes regarding emotional well-being without direct assessment of CDS impact on professionals’ emotional well-being including attitude and perspective on CDS, willingness to perform CDS, dealing with CDS; studies measuring emotional well-being as long-term emotional impairment like depression (v) caregivers were not formal medical professionals, such as social workers, chaplains, family members, relatives and friends.

Based on inclusion and exclusion criteria the study selection was completed by two reviewers (S.Z. and H.M.) independently screening titles and abstracts and than exploring eligibility of full-text articles. Disagreements were resolved through discussion and consensus or arbitration through a third review team member (M.P.).

### Data extraction and quality assessment

For the quality assessment we used a multi-method assessment tool proposed by Hawkers et al. [33]. We evaluated the methodological quality of each study for nine areas: title and abstract, introduction and aims, method and data, sampling, data analysis, ethics and bias, results, transferability and generalizability, and implications and usefulness. Each part was scored as very poor, poor, fair, or good. To complete final ratings, we used supplementary checklists according to cross-sectional (STROBE statement) and qualitative study design (CASP checklist) [34, 35]. Due to the diversity of study design, methodology and outcome measures, a pooled estimate of effect was not calculated.

Using the GRADE approach we assessed the quality of evidence at the outcome level [36]. We evaluated the confidence in estimates of healthcare professionals‘emotional burden with the practice of CDS for physicians and nurses separately. Rating the risk of bias, imprecision, heterogeneity and applicability of the outcomes, we appraised the body of evidence. Each of these four factors could lower the quality of evidence in the case of serious (−1) or very serious (−2) reasons. Final judgments on the quality of evidence of estimates could vary between high, moderate, low, and very low. High means that it is unlikely that further studies will change the results for a specific outcome whereas very low means that it is very likely that the results for a specific outcome will change when additional studies become available. The quality of evidence and the reasons for its decisions as well as the magnitude of the effects are shown in a summary of findings table (Table 5).

## Results

### Search results

**Fig. 1:**
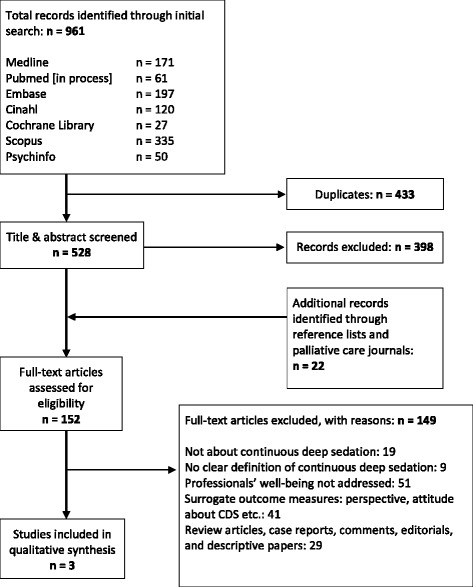
Flow chart of study selection

Initial search yielded 961 records. After removing all duplicates, 528 articles remained for title and abstract screening. Of these we assessed 130 and additional 22 from hand search for full-text eligibility. Three eligible articles remained for quality assessment and data synthesis (Fig. 1).

### Study characteristics

As shown in Table 1, the three studies included two quantitative studies from Japan and one qualitative study from the Netherlands. Data were collected retrospectively by nationwide cross-sectional surveys or semi-structured interviews within different care settings. The three studies analysed a total of 3′900 healthcare professionals comprised of 82% nurses and 18% oncologists or palliative care physicians. Japanese physicians on average had 16 years of experience in oncology and 72% reported less than a quarter of their working time being dedicated for palliative care [37]. Japanese nurses with experience in CDS had a median of 8 sedated patients per year and on average 11 years of clinical experience [38]. In comparison 67.5% of the Dutch nurses had work experience in palliative care or ICU care for more than 5 years [39]. In all study populations the vast majority of patients were cancer patients.

**Table 1:** Study characteristics

First Author, Year	Country	Study Design	Data collection	Setting	Respondents	N enrolled/N analyzed	Patient Diagnosis	Definition of CDS	Burden-related Measurement	Related Case of CDS
Morita T, 2002 [37]	Japan	Quantitative study, nationwide, cross-sectional, retrospective	Questionnaire	Cancer center/Hospital Hospice/PCU	Oncologists^a^ (80%) PC Physicians^b^ (13%)	1436/697	100% Cancer	*“The continuous administration of sedatives to the point where the patient almost (completely) loses consciousness for symptom relief with the primary aim that the patient does not experience suffering. For example, the continuous 24-h administration of 1A (10 mg) of midazolam (Dormicum®) increased to levels such that the patient is almost unconscious”*	Maslach Burnout Inventory: Personal feelings and attitudes on 22 items [0 never experiencing; 6 every day] Three subscales: Emotional exhaustion, 9 items [0–54] Personal accomplishment, 8 items [0–48] Depersonalization, 5 items [0–30]	1) Actual clinical experiences of sedation for cancer patients whose survival was estimated to be 6 months or less 2) Physicians treatment choice for four vignettes [1 unthinkable; 4 strong possibility]
Morita T, 2004 [38]	Japan	Quantitative study, national wide, cross-sectional, retrospective	Questionnaire	Cancer center Hospital PCU	Oncology Nurses^c^ (72%) PC Nurses^c^ (17%)	4210/3187	100% Cancer	*“Continuous use of sedative medications to relieve intolerable and refractory distress by achieving almost or complete unconsciousness until death.”*	One Statement: *“Wish to leave the current work situation due to sedation-related burden” [never, seldom, occasionally, often, always]* Four statements: *“Intensity of negative feelings about sedation practice” [1 strongly disagree; 7 strongly agree]* *Overall burden score:* *Mean score of the four statements about negative feelings*	Overall emotional burden, not patient-specific
Rietjens JAC, 2007 [39]	Netherlands	Qualitative study, retrospective	Semi-structured Interview	PCU MICU	PC Nurses^c^ (62.5%) MICU Nurses^c^ (37.5%)	110/16	75% Cancer 12.5% ALS 6.25% Chronic disease 6.25% Attempted suicide	*“The use of continuous iv. benzodiazepines, barbiturates or other medications to bring an imminently dying patient into a state of unresponsiveness to alleviate suffering from symptoms that cannot be controlled with conventional therapies.”*	Semi-structured Interview: *“Feeling uncomfortable with the use of palliative sedation”*	A memorable case: A case that stuck with you, that made a lasting impression on you, one that you still remember

Note*.* PCU = Palliative Care Unit; MICU = Medical Intensive Care Unit; PC = Palliative Care; CDS = Continuous deep sedation; ALS = Amyotrophic lateral sclerosis

^a^Physicians working at cancer center or general hospitals

^b^Physicians working at hospices or palliative care units

^c^Nurses with experience in CDS

The assessment of the primary endpoint varied across studies using standardized questionnaires or open-ended questions. Two studies addressed emotional well-being to patient-specific CDS and one study focused on perceived emotional burden related to sedation practice. Emotional burden of Japanese physicians was examined using the Maslach Burnout Inventory, which measures the degree of emotional exhaustion (9 items, score range 0–54), depersonalization (8 items, score range 0–48), and lack of personal accomplishment (5 items, score range 0–30) [37]. Higher scores indicate higher levels of the respective subscale. As the authors only reported on factors significantly related to physicians’ treatment choice for physical and existential suffering, there were no results available for physicians’ overall burnout or lack of personal accomplishment scores. Japanese nurses’ emotional burden was assessed independent of specific patient conditions. The questionnaire included one question about the frequency of nurses’ desire to leave current work due to CDS-related burden and four items on nurses' intensity of negative feelings about CDS practice [38]. The mean score of the four items was calculated as overall burden score. Dutch nurses’ burden levels were assessed using semi-structured interview questions about feeling uncomfortable with the use of CDS.

The secondary endpoint regarding factors potentially contributing to CDS-related burden was assessed using open-ended questionnaires. CDS-related concerns of Japanese physicians were measured with 9 of 11 statements about opinions on palliative sedation therapy using a 5-point Likert-type scale of agreement [37]. Japanese nurses had to answer 18 statements about factors contributing to nurses-perceived burden on a 7-point Likert-type scale [38].

For details of study population see Appendix 2.

### Study quality

Quality ranged within and between studies (Table 2). The methodological study quality was determined by two factors: 1) lack of a unique valid and reliable assessment instrument for the outcome of emotional well-being 2) poor control of confounding including high risk for residual confounding. Except for Maslachs Burnout Inventory, the process of questionnaire validation was either absent or reduced to pilot testing including face validity and acceptability. All included studies were retrospective, potentially leading to recall bias. The small response rate in Morita et al. [37] and Rietjens et al. [39] might indicate a selected sample that is not representative for all healthcare professionals involved in inpatient CDS decisions and administration. The cross-sectional design of the quantitative studies only provided information about professionals’ actual emotional well-being and do not allow to consider long-term associations.

**Table 2:** Quality assessment of included studies

	*Title & abstract*	*Introduction & aims*	*Method & data*	*Sampling*	*Data analysis*	*Ethics & bias*	*Results*	*Transferability & generalizability*	*Implications & usefulness*	*Comments*
Morita T, 2002 [37]	++	+	+	+	-	+	+	+	+	Data analysis: low response rate, missing data not explained, no information about overall burnout score
Morita T, 2004 [38]	+	+	+	++	-	+	+	+	++	Validity of endpoints: Single item endpoint; no explanation for cut-off value differentiation low and high-level burden Confounding: Stressors others than CDS influencing nurses burden
Rietjens JAC, 2007 [39]	+	+	-	-	++	++	+	-	++	Recall bias: Memorable case up to 5 years in the past Generalizability: Context information insufficient, no information how context relates to findings; no reference for interview guide; no information about data saturation

*Note*. ++ good; + fair; − poor; −− very poor

### Prevalence of CDS-related emotional burden

As shown in Table 3 in Japan, physicians choosing CDS as strong possibility to treat refractory symptoms, showed medium levels of emotional exhaustion from 19.8 to 20.8 and low levels of depersonalization between 4.55 and 4.21 [37]. These physicians were significantly more emotionally exhausted than those not choosing CDS as a treatment option. The more emotionally exhausted physicians were, the more likely they chose CDS as a possible treatment option for physical (OR 1.02; 1.01 – 1.04) and psychological (OR 1.02; 1.00 – 1.04) refractory symptoms [37] (Appendix 3).

**Table 3:** The prevalence of healthcare professionals’ emotional burden associated with the practice of CDS

First Author, Year	Assessment	Burden-related Measurement		N (100%)	Prevalence
Morita T, 2002 [37]	Questionnaire	Physicians’ mean Burnout-scores in case of physical refractory symptoms		697	Mean, SD
			Emotional exhaustion^a^ in physicians choosing CDS as strong possibility	97	20.8, 11.3
			Emotional exhaustion^a^ in physicians *not* choosing CDS as strong possibility	590	17.5, 10.08
			Depersonalization^b^ in physicians choosing CDS as strong possibility	97	4.55, 4.63
			Depersonalization^b^ in physicians *not* choosing CDS as strong possibility	590	3.58, 4.04
		Physicians’ mean Burnout-scores in case of existential suffering		697	Mean, SD
			Emotional exhaustion^a^ in physicians choosing CDS as strong possibility	102	19.8, 11.6
			Emotional exhaustion^a^ in physicians *not* choosing CDS as strong possibility	576	17.6, 10.8
			Depersonalization^b^ in physicians choosing CDS as strong possibility	102	4.21, 4.72
			Depersonalization^b^ in physicians *not* choosing CDS as strong possibility	576	3.62, 4.01
Morita T, 2004 [38]	Questionnaire	Nurses’ wish to leave the current work situation due to sedation-related burden		2607	%(N)
			Always		0.7 (18)
			Often		3.7 (97)
			Occasionally		26.0 (666)
	Questionnaire	Nurses’ intensity of negative feelings related to CDS		2607	%(N)
			Being involved in sedation is a burden		12 (321)
			Feeling helpless when patient received sedation		11 (313)
			Would avoid performance of sedation if possible		11 (277)
			Feeling that what they had done was of no value when they performed sedation		4.1 (106)
	Questionnaire	Nurses’ overall burden related to CDS^c^		2607	%(N)
			Low-level burden		85.8 (2238)
			High-level burden		14.2 (369)
Rietjens JAC, 2005 [39]	Semi-structured	Nurses’ negative feelings with the use of CDS		16	*n*
	interview		Feeling uncomfortable working on the fine line between CDS and euthanasia		5
			Feeling uncomfortable with the use of CDS for non-physical suffering		4

*Note. M* Mean; *SD* standard deviation; *CDS* continuous deep sedation until death

^a^Burden score range 0-54. Higher scores indicate higher levels of emotional exhaustion

^b^Burden score range 0-30. Higher scores indicate higher levels of depersonalization

^c^Burden score was calculated as the mean score of the four items of negative feelings about CDS, Cronbach's alpha coefficient, 0.86; range 1-7. High-level burden indicates burden score 4.0 or higher; low-level burden indicates burden score below 4.0

With regard to Japanese nurses, one out of 10 nurses reported negative feelings related to the involvement in CDS practice (12%, *n* = 321), feeling helpless (11%, *n* = 313) and avoiding CDS performance (11%, *n* = 277) [38]. High-level burden was prevalent in 14.2% of nurses. Despite the relatively low levels of emotional burden, every fourth nurse (26%, *n* = 666) wanted to leave the current work situation occasionally due to sedation-related burden.

Negative feelings about the use of CDS have also been reported in the qualitative study of Rietjens et al. 2005. Four nurses felt uncomfortable with the use of CDS in case of non-physical suffering and 5 nurses felt distressed for using CDS inappropriately in the context of possible life-shortening end-of-life decisions [39].

### Factors potentially contributing to CDS-related burden

In Japan, almost every second physician and every third nurse had difficulties in accurately determining medical indications and diagnosing refractory symptoms (Table 4). Particularly less experienced nurses reported difficulty accurately determine medical indications and differentiating CDS from possible life-shortening end-of-life decisions [39].

**Table 4:** Prevalence of factors contributing to CDS-related burden in healthcare professionals

First Author, Year	Assessment	Factor	N	%(*n*)
Morita T, 2002 [37]				
	Questionnaire	Physicians’ concerns when performing CDS	697	
		It's difficult to accurately determinate medical indications for CDS^a^		48 (332)
		Associations with the risk to shorten life^a^		37 (260)
		There's a high risk of sedation being performed inappropriately^a^		25 (175)
		Insufficient alleviation of patients suffering^a^		19 (134)
		Difficulties to distinguish CDS from acts to hasten death^a^		17 (119)
		Possibility that less effort would be made for necessary palliative care if the use of CDS became widespread^a^		14 (95)
		Being criticized by the law^a^		12 (81)
		Being criticized by colleagues^a^		5.4 (38)
		Losing patient trust^a^		1.6 (11)
Morita T, 2004 [38]	Questionnaire	Factors contributing to nurses’ perceived burden	3187	
		Frequent experience of unclear patient wishes^b^		29 (768)
		Insufficient time^b^		27 (712)
		Belief that it is difficult to diagnose refractory symptoms^b^		27 (709)
		Nurse-perceived inadequate interpersonal skills^b^		26 (685)
		Nurses-perceived inadequate coping with own grief^b^		11 (281)
		Lack of common understanding of sedation between physicians and nurses^b^		8.1 (211)
		Frequent experience of conflicting wishes between patient and family^b^		8.1 (211)
		Belief that sedation would hasten death^b^		7.2 (187)
		Belief that sedation is indistinguishable from euthanasia^b^		5.4 (142)
		Nurses' personal values contradictory to sedation^b^		4.1 (107)
		Team conference unavailable^b^		2.1 (132)
Rietjens JAC, 2007 [39]	Semi-structured interview	Feeling uncomfortable using CDS for nonphysical symptoms	16	
		Experience with CDS		Inverse relation
	Semi-structured interview	Feeling uncomfortable working on the fine line between CDS and euthanasia	16	
		Experience with CDS		Inverse relation

*Note*. Numbers and percentages refer to those who agree or strongly agree to respective statement. CDS = Continuous deep sedation

^a^Rated as the degree of agreement from 1 (strongly disagree) to 5 (strongly agree)

^b^Rated as the degree of agreement from 1 (strongly disagree) to 7 (strongly agree)

Multivariate analyses revealed that CDS-related burden is more likely in nurses with little clinical experience, which perceive CDS as contradictory to their own values and have difficulty coping with their own grief. Less years of clinical experience were associated with a 2% increase in the probability of the desire to leave current work (OR .98; 95% CI .96 – .99) [38]. In turn, for each year of additional clinical experience Japanese nurses’ burden score decreased by .06 (*β* = −.06; −.01 – -.00) [38]. For details of multivariate analyses see Appendix 3.

The most frequent non-clinical factors medical staff is confronted with, were unclear patient wishes (29%), disagreements between patient and family (17%) and time pressure (27%). Nurses reported marginally higher CDS-related burden in the absence of regular team meetings (*β* = .07; .04 – .10) and frequent conflicting wishes between patient and family (*β* = .05; .02 – .09) [38]. The risk of reporting a desire to leave current work due to CDS was 11% higher among those reporting conflicting wishes between patient and family (OR 1.11; 1.02 – 1.22), 17% higher among those reporting a lack of common understanding of sedation with physicians (OR 1.17; 1.09 – 1.26) and 9% higher among those reporting lack of team conferences (OR 1.09; 1.01 – 1.18) [38].

### Quality of the evidence

RCTs are considered as “gold standard” for interventions, as randomization reduces confounding. Our research questions focus on burden of disease and possible determinants and not on interventions. Therefore we judged well-designed observational studies as high quality of evidence. For details of quality of GRADE ratings see Appendix 4.

The confidence in estimates for healthcare professionals’ emotional burden related to the practice of CDS was low. The main reasons for downgrading the confidence in estimates were due to study limitations and heterogeneity of results. Healthcare professionals’ burden related to the practice of CDS was assessed using single item questions and heterogeneity was already given within studies. Furthermore, outcomes from different care settings and professional groups, e.g., oncologists and palliative care specialists, were analysed jointly. The assessment of CDS-related emotional burden differed between overall burden levels independent of patient conditions and patient-specific emotional burden of healthcare professionals. In case of patient-specific burden there might be recall bias. Across eligible studies healthcare professionals’ emotional burden was self-reported and might be influenced by confounders leading to emotional distress in general. Where CDS-related burden was high it seems possible that healthcare professionals already left their current work place and therefore selection bias might have occurred. Multivariate analyses are limited to Japanese healthcare professionals working in the oncology setting including their cultural and legal background. For summary of findings see Table 5.

**Table 5:** Summary of findings

What is the effect of the inpatient practice of CDS on healthcare professionals’ emotional well-being?						
Population: Healthcare professionals practicing CDS Setting: Hospital, hospice, palliative care unit, cancer centre Intervention: Continuous deep sedation until death for terminally ill adult patients (≥18 years age) Outcome: Healthcare professionals’ emotional burden						
Endpoints	Prevalence	Specific indicators	Effect of estimates (95% CI)	Participants^a^ (N, studies)	Confidence in estimates^b^	Comments
CDS-related burden in physicians [37]	Emotional exhaustion [M, SD]: - Physicians choosing CDS as treatment option for refractory dyspnoea: 20.8, 11.3 - Physicians *not* choosing CDS as treatment option for refractory dyspnoea: 17.6, 10.8		Comparison of physicians emotional exhaustion depending on treatment choice: *p* < 0.01	697, 1	++oo low	Physicians’ emotional exhaustion is an independent determinant for choosing CDS as treatment option for refractory dyspnoea: OR = 1.02 (1.01;1.04) *p* = 0.14
		9 concerns about CDS [Agreement on 11 statements about CDS]	Range 1.6% concerns losing patients trust to 48% reporting difficulties to accurately determinate medical indications for CDS			Only descriptives, not assessed in multivariate models as independent determinants of physicians’ emotional burden.
CDS-related burden in nurses [38, 39]	Burden score^c^ [%, N(100)]: 14.2%, 2607	11 independent determinants	Strongest effect: Nurses’ personal values contradictory to CDS β = 0.27 (.24;.30)^d^	3203, 2	++oo low	
	Desire to leave work occasionally [%, N(100)]: 26%, 2607	11 independent determinants	Strongest effect: Nurse-perceived inadequate coping with own grief OR = 1.23 (1.14;1.32)^e^			
	Feeling uncomfortable working on the fine line between CDS and euthanasia *n* = 5	Clinical experience	-			*N* = 16
	Feeling uncomfortable with the use of CDS for non-physical suffering *n* = 4	Clinical experience	-			*N* = 16

*Note.* CDS = Continuous deep sedation; OR = Odds ratio; CI = Confidence interval

^a^Total number of participants analysed

^b^GRADE Working Group grades of evidence. High quality: Further research is very unlikely to change our confidence in the estimate of effect. Moderate quality: Further research is likely to have an important impact on our confidence in the estimate of effect and may change the estimate. Low quality: Further research is very likely to have an important impact on our confidence in the estimate of effect and is likely to change the estimate. Very low quality: We are very uncertain about the estimate

^c^Burden score was calculated as mean score of four items, Cronbach’s α 0.86; the higher the score means the higher nurses burden

^d^β values for linear regression analyses using burden score as dependent variable. F = 76, *p* < .001, R2 = 0.24

^e^Odds ratios for logistic regression analyses comparing the nurses who wanted to leave current work occasionally, often or always and others

## Discussion

To the best of our knowledge this is the first systematic review investigating the evidence of the effect of continuous deep sedation until death on healthcare professionals’ emotional well-being. Across included studies, the prevalence of CDS-related emotional burden varies widely; while the effect is small in magnitude, it may seriously impact professionals to the point that they want to leave their current work. Prevalent concerns contributing to CDS-related burden were difficulty in diagnosing refractory symptoms, sedation in the context of life-shortening decisions, conflicting wishes between patients and families, disagreements within the care team, and inadequate skills and coping among healthcare professionals. Higher emotional burden was more likely when healthcare professionals were confronted with these clinical and non-clinical challenges, making CDS justification difficult. This appeared to be in part a function of clinical experience. These findings are not yet generalizable to all healthcare professionals close to inpatient CDS decisions and administration as the small number of included studies is restricted to two countries and includes almost entirely cancer patients. The retrospective design may introduce recall bias and the low response rates in Japanese physicians and Dutch nurses imply a selected sample not necessarily representative for all professionals involved in CDS practice. Therefore, the quality of evidence for healthcare professionals’ emotional burden is low and it is likely that additional studies will change the current evidence base.

This systematic review revealed that there is little evidence about CDS-related emotional burden in healthcare professionals. Despite the increasing trends of CDS practice, only three studies could be identified. It is hardly surprising that these were either Dutch or Japanese, as bibliometric analysis has shown that best evidence of CDS research activity originated in the Netherlands and Japan [40]. Across the reviewed studies no shared target outcome measure for professionals’ well-being was available. This indicates that there is a lack of a common understanding about the mechanism of how the practice of CDS is related to healthcare professionals’ emotional well-being. Raus et al. [27] have shown that the emotional impact of being involved in CDS depends on different dimensions of closeness, defined as the degree to which healthcare professionals feel responsible for CDS decisions or administration [28]. Accordingly, the risk for CDS-related burden is related to professionals’ perceived closeness in CDS decisions, their emotional and physical closeness to the patient, and their perceived causal closeness [28]. Causal closeness is defined as healthcare professionals’ feeling close to sequences of events, such as CDS administration and the patient’s death [28]. The more causal closeness professionals perceive the more morally responsible they feel [28]. The degree of professionals’ emotional involvement and physical closeness to patient’s care, their decision making authority, and the perceived causal closeness vary considerably between the professional’s role, their work setting, and the cultural and legal background. This could partly explain the high heterogeneity of CDS-related burden across included studies.

We have found different CDS-related burden prevalence for nurses and physicians. These results are consistent with surrogate outcome measures about attitudes towards CDS. Despite predominantly positive experiences there is high variability between healthcare professionals [20]. According to Swart et al. [41], physicians more often felt pressured to start CDS whereas nurses have reported more concerns regarding CDS and death hastening [41]. Nurses have a supportive role in decision-making, but an active one in CDS administration and monitoring [42]. By caring for a patient they are emotionally and physically closer to patient and relatives [28, 42]. In turn, physicians have more decisional authority and responsibility [28, 42]. These role models could partly explain the different degrees of closeness and therefore the variability in CDS-related burden between healthcare professionals.

CDS-related burden varies not only between healthcare professionals but also between countries. According to Seymour et al. [43] perspectives and attitudes towards CDS depend on healthcare professionals’ cultural and legal background. Compared with the Netherlands and Belgium, professionals from the UK showed more cautious attitudes and struggled more often with differentiating CDS from death hastening [43]. In the Netherlands and Belgium, CDS is more often based on formal medical decision-making according to specific CDS guidelines [43]. It seems that established guidelines contribute to rather homogenous and convinced attitudes towards CDS by reducing causal closeness through resolving misconceptions about CDS and life-shortening practices [28]. Established clinical guidelines about CDS decision-making, indication, and administration seem particularly important, as misconceptions cannot only be resolved through a universal CDS definition.

We have found that misconceptions about CDS are likely present regardless of a universal CDS definition. Approximately 10% of Japanese nurses reported a lack of common CDS understanding despite a unique CDS definition. This highlights that a universal terminology is not sufficient for a common understanding of CDS [38]. Although the most recent term used is palliative sedation, current definitions vary in terms of indication, patient populations, medications, initiation, and ethical considerations [44]. Papavasiliou and colleagues 2013 have demonstrated that this heterogeneity in definition is not only due to terminology itself. Rather, it seems that there is a different language and vocabulary used within palliative care [44].

Our findings indicate that professionals’ clinical experience is an important factor contributing to a common understanding of CDS definition and indication. A common understanding of CDS is particularly important considering the increased risk of CDS-related burden by its lack of distinction from possibly life-shortening decisions. Foley et al. 2015 reveal that a higher degree of palliative care specialization and CDS experience correlates with physician’s certainty that CDS is not life shortening [45]. In contrast, less experienced physicians working in unspecialized settings are more likely to consider CDS as death hastening and either avoid its use or report ambiguous attitudes [45]. Similar results are available for nurses [46, 47]. Nurses are more prepared to administer CDS with higher education, more end-of-life care experience, and when working in hospital [47].

We demonstrate that beside CDS terminology and understanding, inconsistencies are particularly present in describing patients’ level of suffering. It remains challenging to decide when a symptom is refractory and unbearable as this is both a physician’s and a patient’s decision [21]. Therefore, CDS decision-making should be based on multidisciplinary decisions involving the patient and families. There is evidence that healthcare professionals’ experience with the practice of CDS is particularly positive when decisions are made in a team involving patients and the family [47–50]. Recent findings have confirmed that the process of multidisciplinary decision-making aids professionals dealing with the CDS practice by reducing its emotional impact through shared responsibility [27]. As a source of emotional support an effective team approach can even protect palliative care professionals from burnout [14, 51–53]. Despite promising burnout prevention, there is still no standard psychological intervention for palliative care staff, as the variety in measures to assess professionals’ well-being makes it hard to chose a common target outcome [54, 55].

The results of this systematic review highlight the importance of a common understanding of CDS and further education and training to improve professionals’ experience and personal skills. There is a need for a greater understanding of cultural differences in ethical perspectives on CDS practice as professionals’ values and concerns differ between countries [43]. With regard to Abarshi and colleagues’ checklist for the quality appraisal of palliative sedation guidelines, international guidelines should focus more on the decision-making process taking into account the differences in CDS practice including professionals’ roles and attitudes, and emphasize the clear distinction of CDS and possibly life-shortening end-of-life decisions [56, 57].

### Strength and potential bias in the review process

To the best of our knowledge this is the first systematic review addressing the impact of the practice of CDS on healthcare professionals’ emotional well-being. We followed a predefined review protocol and standard systematic review methodology. A limitation may be that we had to accept any measure of emotional well-being as there exists no standard metric. We conducted a broad search strategy to cover the topic of emotional well-being but however we might have missed some studies since search terms may be somewhat ambiguous. We ensured to include all studies using a measure of emotional well-being that is associated with the practice of CDS. The decision to exclude surrogate outcome measures is indicative of the trade-offs necessary for a review to evaluate high quality evidence.

### Research needs

There is need for methods to evaluate professionals’ emotional well-being related to the practice of CDS. A standardized reliable and valid instrument should be provided to assess emotional well-being taking into account the complex context including CDS-related turnover. Future research should examine if overall burden or burden related to specific patient conditions is more valid. Healthcare professionals are exposed to diverse work environments, patient disease groups, and goal focus including palliation and curative care. Therefore, studies should look at professionals’ burden of different clinical settings separately considering the care roles, the opportunity for multidisciplinary decision-making, and the patient population. Additionally comparative studies exploring the influence of guideline use, particularly in terms of determination of CDS indication and decision-making should be conducted. Cultural differences including the legal background of CDS, of physician-assisted suicide and euthanasia might influence healthcare professionals’ attitude towards the practice of CDS and should be considered in the future.

## Conclusions

Current evidence does not suggest that the practice of CDS is generally associated with lower emotional well-being of healthcare professionals but clinical and non-clinical challenges of CDS decision-making appears to play a critical role. There is an increased risk of emotional distress when healthcare professionals struggle with clinical and ethical justifications for CDS performance what in turn seems related with professionals’ clinical experience and CDS knowledge.

## Appendix 1

**Table 6:** Search strategy

*Search in Medline (via Ebsco)* *Date of search: 6th October 2016*	
*End-of-life*	
# 1	MM "Palliative Care" OR MM "Palliative Medicine"
# 2	MM "Hospice Care" OR MM "Hospice and Palliative Care Nursing"
# 3	MH "Terminal Care+" OR MM "Terminally Ill"
# 4	/OR #1 - #3
# 5	(palliat* OR terminal* OR "end-of-life" OR hospice) N3 care
# 6	#4 OR #5
*Sedation*	
# 7	MM "Conscious Sedation" OR MM "Deep Sedation"
# 8	(palliat* OR continuous* OR terminal OR deep OR total OR "end-of-life") N3 sedat*
# 9	#7 OR #9
*Emotional well-being*	
# 10	MH "Emotions+"
# 11	MH "Stress, Psychological+"
# 12	MH "Attitude of Health Personnel+"
# 13	MM "Occupational Health Nursing" OR MM "Occupational Health Physicians" OR MM "Occupational Health"
# 14	MM "Job Satisfaction"
# 15	MM "Adaptation, Psychological"
# 16	/OR #10 - #15
#17	(wellbeing OR "well-being" OR exhaustion OR burden OR health) N3 emotion*
# 18	#16 OR #17
# 19	#6 AND #9 AND #18
	Hits: 171

*Note*. This initial search strategy was adapted to Pubmed, Embase, Cochrane Library, Cinahl, Scopus and PsycINFO

Overall search cleaned from duplicates yielded 528 records

## Appendix 2

**Table 7:** Study population

First Author, Year	Profession	N analyzed	Setting % (N)	Age (Mean, SD)	Sex (% male/female)	Religion % (N)	Clinical experience, Years (Mean, SD)	Palliative Care experience	Experience with CDS
Morita T, 2002 [37]	Oncologists Palliative Care Physicians	697	CC and Hospital: 80 (560) Hospice and PCU: 13 (87)	45, 8.2	92/6.2	Buddhism: 16 (108) Christianity: 6.7 (47) Others: 1.9 (13) None: 75 (521)	Oncology experience: 16, 8.1	Time used for palliative care: 72% used less than 25% of their working time	No. of physicians who used CDS for physical suffering: *n* = 456, 66% No. of physicians who used CDS for psychological suffering: *n* = 262, 38%
Morita T, 2004 [38]	Oncology Nurses Palliative Care Nurses	2607^a^	CC: 46 (1197) General Hospital: 33 (868) PCU: 20 (525)	33, 8.6	1/99	n.r.	Clinical experience: 11, 8.4	n.r.	No. of patients they cared for during the previous year who received CDS: Median of 8 patients/year
Rietjens JAC, 2007 [39]	Palliative Care Nurses Intensive Care Nurses	16^a^	PCU: 62.5 (10) MICU: 37.5 (6)	38, n.r.	6.2/93.8	Roman Catholic: 43.7 (7) Protestant: 12.5 (2) Others: 31.3 (5) None: 12.5 (2)	n.r.	Work experience in palliative care or ICU care: < 5 years: 6 (37.5%) 5-10 years: 4 (25%) > 10 years: 6 (37.5%)	n.r.

*Note. CC* Cancer Center, *PCU* Palliative Care Unit, *MICU* Medical Intensive Care Unit, *ICU* Intensive Care Unit, *CDS* Continuous deep sedation, *SD* Standard deviation, *n.r.* Not reported

^a^Nurses with experience in CDS

## Appendix 3

**Table 8:** Multivariate analysis of factors contributing to CDS-related burden in healthcare professionals

First Author, Year		Covariate		Measure of Association	*p* Value
Morita T, 2002 [37]	Logistic Regression^a^	Physicians’ decision to choose CDS as treatment option		OR (95% CI)	
		1) For refractory dyspnoea			
			Physicians' preference for their own end-of-life care	1.53 (1.07, 2.20)	0.021
			Emotional exhaustion	1.02 (1.01, 1.04)	0.014
		2) For existential suffering			
			Emotional exhaustion	1.02 (1.00, 1.04)	0.060
Morita T, 2004 [38]	Logistic Regression^b^	Nurses’ desire to leave current work (N = 369)		OR (95% CI)	
			Clinical experience (years)	0.98 (0.96,0.99)	<.001
			Insufficient time^c^	1.17 (1.10, 1.25)	<.001
			Lack of common understanding of sedation between physicians and nurses^c^	1.17 (1.09, 1.26)	<.001
			Team conference unavailable^c^	1.09 (1.01, 1.18)	0.021
			Frequent experience of conflicting wishes between patient and family^c^	1.11 (1.02, 1.22)	0.014
			Nurse-perceived inadequate interpersonal skills^c^	-	ns
			Belief that it is difficult to diagnose refractory symptoms^c^	1.11 (1.03, 1.20)	<.001
			Belief that sedation would hasten death^c^	-	ns
			Belief that sedation is indistinguishable from euthanasia^c^	1.15 (1.07, 1.23)	<.001
			Nurses-perceived inadequate coping with own grief^c^	1.23 (1.14, 1.32)	<.001
			Nurses' personal values contradictory to sedation^c^	1.14 (1.06, 1.23)	<.001
	Linear Regression^d^	Nurses’ overall burden (N = 369)		Beta (95% CI)	
			Clinical experience (years)	-0.062 (-0.011,-0.002)	0.009
			Insufficient time^c^	0.029 (0.005, 0.053)	0.019
			Lack of common understanding of sedation between physicians and nurses^c^	-	ns
			Team conference unavailable^c^	0.066 (0.035, 0.097)	<.001
			Frequent experience of conflicting wishes between patient and family^c^	0.051 (0.017, 0.085)	0.003
			Nurse-perceived inadequate interpersonal skills^c^	0.060 (0.026, 0.093)	<.001
			Belief that it is difficult to diagnose refractory symptoms^c^	0.073 (0.041, 0.11)	<.001
			Belief that sedation would hasten death^c^	0.057 (0.028, 0.085)	<.001
			Belief that sedation is indistinguishable from euthanasia^c^	0.054 (0.023, 0.085)	<.001
			Nurses-perceived inadequate coping with own grief ^c^	0.075 (0.043, 0.11)	<.001
			Nurses' personal values contradictory to sedation^c^	0.27 (0.24, 0.30)	<.001

*Note. CDS* Continuous deep sedation; *OR* Odds ratio; *CI* Confidence interval; *n.s.* not significant

^a^Multiple logistic regression analysis comparing physicians who chose CDS as strong possibility with others. Physicians’ preference for symptomatic treatment was assessed using 3 items, score range of 1 to 5 (higher preference); emotional exhaustion was assessed using a subscale of Maslach Burnout Inventory, Cronbach's alpha range 0.6 to 0.88, score range of 0 to 54 (high exhaustion)

^b^Logistic regression comparing nurses who wished to leave the current work occasionally, often, or always and others

^c^Rated as the degree of agreement on each statement from 1 (strongly disagree) to 7 (strongly agree)

^d^Multiple linear regression only included nurses with high-level emotional burden, F = 76, p < .001, R2 = 0.24; Burden score was calculated as the mean score of four items about CDS-related negative feelings, Cronbach's alpha coefficient 0.86; the higher score means the higher nurses burden

## Appendix 4

**Table 9:** Confidence in estimates of healthcare professionals’ emotional burden associated with the practice of CDS

Outcome	No of studies	Risk of bias	Imprecision	Heterogeneity	Applicability	Confidence^a^
		0	0	−1	−1	+ooo; low
CDS-related burden in physicians	1, Morita T, 2002 [37]	Confounding: Physicians’ concerns regardless of patients characteristics and other confounders	Physicians’ burden related to choose CDS as treatment option for specific vignettes Low response rate 49.6%	Variability in mean scores within and between Maslach Burnout Inventory subscales of emotional exhaustion and depersonalization Variability in prevalence of physicians’ concerns ranging from 1.6-48%	Population: Japanese oncologists and palliative care specialists with experience in sedation for cancer patients Intervention: CDS for cancer patients in vignettes with family support and clinical survival prediction of less than 3 weeks Comparator: Physicians choosing CDS as strong treatment option vs. others Outcome: Self-reported Not clear, if patient-specific No long-term associations possible	
		−1	0	−1	0	+ooo; low
CDS-related burden in nurses	2, Morita T, 2004 [38] Rietjens JAC, 2007 [39]	Study limitations: Validity of outcome measures Confounding: Stressors influencing general wish to leave current work, R^2^ = .24 Selection Bias: Study does not include burdened nurses who already left their current work due to CDS Recall Bias: A memorable case up to 5 years in the past	Nurses’ wish to leave current work as single item questions	Heterogeneity in targeted psychological outcome Variability in prevalence on single item level and across outcomes	Population: Japanese & Dutch oncology and palliative care nurses with experience in the practice of CDS Intervention: CDS for cancer patients predominantly Comparator: Nurses with low-level burden vs. high-level burden Outcome: Self-reported Overall burden No long-term associations possible	

Note. *CDS* Continuous deep sedation

^a^GRADE Working Group grades of evidence. High quality: Further research is very unlikely to change our confidence in the estimate of effect. Moderate quality: Further research is likely to have an important impact on our confidence in the estimate of effect and may change the estimate. Low quality: Further research is very likely to have an important impact on our confidence in the estimate of effect and is likely to change the estimate. Very low quality: We are very uncertain about the estimate

## Abbreviations

ALS: Amyotrophic lateral sclerosis
CASP checklist: Critical Appraisal Skills Programme
CC: Cancer center
CDS: Continuous deep sedation until death
CI: Confidence interval
GRADE approach: Grading of Recommendations Assessment, Development and Evaluation
M: Mean
MICU: Medical intensive care unit
OR: Odds ratio
PCU: Palliative care unit
PRISMA: Preferred Reporting Items for Systematic Reviews and Meta-Analysis
PS: Palliative sedation
SD: Standard deviation
STROBE statement: Strengthening the Reporting of Observational studies in Epidemiology

## References

[1] Cherny NI, Radbruch L (2009). European Association for Palliative Care (EAPC) recommended framework for the use of sedation in palliative care. Palliat Med.

[2] Swart SJ, van der Heide A, van Zuylen L, Perez RSGM, Zuurmond WWA, van der Maas PJ (2012). Considerations of physicians about the depth of palliative sedation at the end of life. CMAJ.

[3] Cherny NI (2014). ESMO Clinical Practice Guidelines for the management of refractory symptoms at the end of life and the use of palliative sedation. Ann Oncol.

[4] Schildmann EK, Schildmann J, Kiesewetter I (2015). Medication and Monitoring in Palliative Sedation Therapy: A Systematic Review and Quality Assessment of Published Guidelines. J Pain Symptom Manage.

[5] Reuzel RPB, Hasselaar GJ, Vissers KCP, van der Wilt GJ, Groenewoud JMM, Crul BJP (2008). Inappropriateness of using opioids for end-stage palliative sedation: a Dutch study. Palliat Med.

[6] de Graeff A, Dean M (2007). Palliative sedation therapy in the last weeks of life: a literature review and recommendations for standards. J Palliat Med.

[7] Miccinesi G, Rietjens JAC, Deliens L, Paci E, Bosshard G, Nilstun T (2006). Continuous deep sedation: Physicians’ experiences in six European countries. J Pain Symptom Manage.

[8] Seale C (2010). Continuous deep sedation in medical practice: a descriptive study. J Pain Symptom Manage.

[9] Robijn L, Cohen J, Rietjens J, Deliens L, Chambaere K (2016). Trends in Continuous Deep Sedation until Death between 2007 and 2013: A Repeated Nationwide Survey. PLoS One.

[10] Onwuteaka-Philipsen BD, Brinkman-Stoppelenburg A, Penning C, de Jong-Krul G, van Delden JJM, van der Heide A (2012). Trends in end-of-life practices before and after the enactment of the euthanasia law in the Netherlands from 1990 to 2010: A repeated cross-sectional survey. Lancet.

[11] Bosshard G, Zellweger U, Bopp M, Schmid M, Hurst SA, Puhan MA (2016). Medical end-of-Life practices in Switzerland: A comparison of 2001 and 2013. JAMA Intern Med.

[12] Anquinet L, Rietjens JA, Seale C, Seymour J, Deliens L, van der Heide A (2012). The practice of continuous deep sedation until death in flanders (belgium), the Netherlands, and the u.k.: a comparative study. J Pain Symptom Manag.

[13] Anquinet L, Raus K, Sterckx S, Smets T, Deliens L, Rietjens JAC (2014). Re: A response to Willis et al. Palliat Med.

[14] Perez GK, Haime V, Jackson V, Chittenden E, Mehta DH, Park ER (2015). Promoting resiliency among palliative care clinicians: stressors, coping strategies, and training needs. J Palliat Med.

[15] Yoon JD, Hunt NB, Ravella KC, Jun CS, Curlin FA (2016). Physician Burnout and the Calling to Care for the Dying: A National Survey. Am J Hosp Palliat Med.

[16] Asai M, Morita T, Akechi T, Sugawara Y, Fujimori M, Akizuki N (2007). Burnout and psychiatric morbidity among physicians engaged in end-of-life care for cancer patients: a cross-sectional nationwide survey in Japan. Psychooncology.

[17] Kamal AH, Bull JH, Wolf SP, Swetz KM, Shanafelt TD, Ast K (2016). Prevalence and Predictors of Burnout Among Hospice and Palliative Care Clinicians in the U.S.. J Pain Symptom Manage.

[18] Schildmann J, Schildmann E (2013). Clinical and ethical challenges of palliative sedation therapy. The need for clear guidance and professional competencies. Int J Clin Pract.

[19] Robijn L, Chambaere K, Raus K, Rietjens J, Deliens L (2015). Reasons for continuous sedation until death in cancer patients: A qualitative interview study. Eur J Cancer Care (Engl).

[20] Abarshi EA, Papavasiliou E, Preston N, Brown J, Payne S (2014). The complexity of nurses’ attitudes and practice of sedation at the end of life: A systematic literature review. J Pain Symptom Manage.

[21] Juth N, Lindblad A, Lynöe N, Sjöstrand M, Helgesson G (2010). European Association for Palliative Care (EAPC) framework for palliative sedation: An ethical discussion. BMC Palliat Care.

[22] Gran SV, Miller J (2008). Norwegian nurses’ thoughts and feelings regarding the ethics of palliative sedation. Int J Palliat Nurs.

[23] Rietjens JAC, Buiting HM, Pasman HRW, van der Maas PJ, van Delden JJM, van der Heide A (2009). Deciding about continuous deep sedation: physicians’ perspectives: a focus group study. Palliat Med.

[24] Anquinet L, Raus K, Sterckx S, Smets T, Deliens L, Rietjens JAC (2012). Similarities and differences between continuous sedation until death and euthanasia - Professional caregivers’ attitudes and experiences: A focus group study. Palliat Med.

[25] Maeda I, Morita T, Yamaguchi T, Inoue S, Ikenaga M, Matsumoto Y (2016). Effect of continuous deep sedation on survival in patients with advanced cancer (J-Proval): a propensity score-weighted analysis of a prospective cohort study. Lancet Oncol.

[26] Beller EM, van Driel ML, McGregor L, Truong S, Mitchell G (2015). Palliative pharmacological sedation for terminally ill adults. Cochrane Database Syst Rev.

[27] Raus K, Anquinet L, Rietjens JAC, Deliens L, Mortier F, Sterckx S (2014). Factors that facilitate or constrain the use of continuous sedation at the end of life by physicians and nurses in Belgium: results from a focus group study. J Med Ethics.

[28] Raus K, Brown J, Seale C, Rietjens JAC, Janssens R, Bruinsma SM (2014). Continuous sedation until death: the everyday moral reasoning of physicians, nurses and family caregivers in the UK, The Netherlands and Belgium. BMC Med Ethics.

[29] Van Deijck RHPD, Hasselaar JGJ, Verhagen SCAHHVM, Vissers KCP, Koopmans RTCM (2016). Patient-Related Determinants of the Administration of Continuous Palliative Sedation in Hospices and Palliative Care Units: A Prospective, Multicenter, Observational Study. J Pain Symptom Manage.

[30] Welp A, Meier LL, Manser T (2016). The interplay between teamwork, clinicians’ emotional exhaustion, and clinician-rated patient safety: a longitudinal study. Crit Care.

[31] Centre for Reviews and Dissemination (2009). Systematic reviews: CRD’s guidance for undertaking reviews in health care.

[32] Moher D, Liberati A, Tetzlaff J, Altman DG (2009). Preferred Reporting Items for Systematic Reviews and Meta-Analyses: the PRISMA statements. PLoS Med.

[33] Hawker S, Payne S, Kerr C, Hardey M, Powell J (2002). Appraising the evidence: reviewing disparate data systematically. Qual Health Res.

[34] Vandenbroucke JP, von Elm E, Altman DG, Gotzsche PC, Mulrow CD, Pocock SJ (2007). Checklist of items that should be included in reports of cross-sectional studies. Ann Intern Med.

[35] Critical Appraisal Skills Programm (CASP) - Qualitative Research Checklist. Oxford CASP. 2014. http://media.wix.com/ugd/dded87_29c5b002d99342f788c6ac670e49f274.pdf. Accessed 25 Oct 2015.

[36] Guyatt GH, Oxman AD, Schünemann HJ, Tugwell P, Knottnerus A (2011). GRADE guidelines: a new series of articles in the Journal of Clinical Epidemiology. J Clin Epidemiol.

[37] Morita T, Akechi T, Sugawara Y, Chihara S, Uchitomi Y (2002). Practices and attitudes of Japanese oncologists and palliative care physicians concerning terminal sedation: a nationwide survey. J Clin Oncol.

[38] Morita T, Miyashita M, Kimura R, Adachi I, Shima Y (2004). Emotional burden of nurses in palliative sedation therapy. Palliat Med.

[39] Rietjens JAC, Hauser J, van der Heide A, Emanuel L (2007). Having a difficult time leaving: Experiences and attitudes of nurses with palliative sedation. Palliat Med.

[40] Papavasiliou E, Payne S, Brearley S, Brown J, Seymour J (2013). Continuous sedation (CS) until death: Mapping the literature by bibliometric analysis. J Pain Symptom Manage.

[41] Swart SJ, Brinkkemper T, Rietjens JAC, Blanker MH, van Zuylen L, Ribbe M (2010). Physicians’ and nurses’ experiences with continuous palliative sedation in the Netherlands. Arch Intern Med.

[42] Arevalo JJ, Rietjens JA, Swart SJ, Perez RSGM, van der Heide A (2013). Day-to-day care in palliative sedation: Survey of nurses’ experiences with decision-making and performance. Int J Nurs Stud.

[43] Seymour J, Rietjens J, Bruinsma S, Deliens L, Sterckx S, Mortier F (2015). Using continuous sedation until death for cancer patients: a qualitative interview study of physicians’ and nurses’ practice in three European countries. Palliat Med.

[44] Papavasiliou ES, Brearley SG, Seymour JE, Brown J, Payne SA (2013). From sedation to continuous sedation until death: How has the conceptual basis of sedation in end-of-life care changed over time?. J Pain Symptom Manage.

[45] Foley R-A, Johnston WS, Bernard M, Canevascini M, Currat T, Borasio GD (2015). Attitudes Regarding Palliative Sedation and Death Hastening Among Swiss Physicians: A Contextually Sensitive Approach. Death Stud.

[46] Inghelbrecht E, Bilsen J, Mortier F, Deliens L (2011). Continuous deep sedation until death in Belgium: a survey among nurses. J Pain Symptom Manage.

[47] Inghelbrecht E, Bilsen J, Mortier F, Deliens L (2009). Nurses’ attitudes towards end-of-life decisions in medical practice: A nationwide study in Flanders. Belgium Palliat Med.

[48] van Tol DG, Kouwenhoven P, van der Vegt B, Weyers H (2015). Dutch physicians on the role of the family in continuous sedation. J Med Ethics.

[49] Brinkkemper T, Rietjens JAC, Deliens L, Ribbe MW, Swart SJ, Loer SA (2015). A Favorable Course of Palliative Sedation: Searching for Indicators Using Caregivers’ Perspectives. Am J Hosp Palliat Med.

[50] Rys S, Deschepper R, Deliens L, Mortier F, Bilsen J (2013). Justifying continuous sedation until death: A focus group study in nursing homes in Flanders, Belgium. Geriatr Nurs (Minneap).

[51] Hernandez-Marrero P, Pereira SM, Carvalho AS (2015). Ethical Decisions in Palliative Care: Interprofessional Relations as a Burnout Protective Factor? Results From a Mixed-Methods Multicenter Study in Portugal. Am J Hosp Palliat Care.

[52] Swetz KM, Harrington SE, Matsuyama RK, Shanafelt TD, Lyckholm LJ (2009). Strategies for avoiding burnout in hospice and palliative medicine: peer advice for physicians on achieving longevity and fulfillment. J Palliat Med.

[53] Zambrano SC, Chur-Hansen A, Crawford GB (2014). The experiences, coping mechanisms, and impact of death and dying on palliative medicine specialists. Palliat Support Care.

[54] Hill RC, Dempster M, Donnelly M, McCorry NK (2016). Improving the wellbeing of staff who work in palliative care settings: A systematic review of psychosocial interventions. Palliat Med.

[55] Melo CG, Oliver D (2011). Can addressing death anxiety reduce health care workers’ burnout and improve patient care?. J Palliat Care.

[56] Abarshi E, Rietjens J, Caraceni A, Payne S, Deliens L, Van Den Block L (2014). Towards a standardised approach for evaluating guidelines and guidance documents on palliative sedation: Study protocol. BMC Palliat Care.

[57] Schildmann EK, Bausewein C, Schildmann J (2015). Palliative sedation: Improvement of guidelines necessary, but not sufficient. Palliat Med.

